# Effective production of lactosucrose using β‐fructofuranosidase and glucose oxidase co‐immobilized by sol–gel encapsulation

**DOI:** 10.1002/fsn3.1195

**Published:** 2019-09-05

**Authors:** Jie Long, Ting Pan, Zhengjun Xie, Xueming Xu, Zhengyu Jin

**Affiliations:** ^1^ The State Key Laboratory of Food Science and Technology Jiangnan University Wuxi China; ^2^ School of Food Science and Technology Jiangnan University Wuxi China; ^3^ Collaborative Innovation Center of Food Safety and Quality Control in Jiangsu Province Jiangnan University Wuxi China

**Keywords:** co‐immobilization, glucose oxidase, lactosucrose, stability, β‐fructofuranosidase

## Abstract

The production of lactosucrose is hampered by the costly use of β‐fructofuranosidase, which shows poor stability and a low efficiency in transfructosylation activity. Immobilization could improve enzyme stability and realize the cyclic utilization at a reduced cost. In order to eliminate the by‐product inhibition and improve the transfructosylation efficiency, β‐fructofuranosidase and glucose oxidase were co‐immobilized by sol–gel encapsulation and the subsequent production efficiency of lactosucrose was investigated. The as‐prepared immobilized bi‐enzymes retained 85.39% of their initial activity at an enzyme concentration of 1.47 mg/g·sol during immobilization and showed great operational stability (maintaining 78.5% of their initial activity) after 15 consecutive reuses. The yield of lactosucrose synthesized by immobilized bi‐enzymes reached 160.8 g/L under the optimized conditions, which was relatively higher than previous reported results. Moreover, the yield of lactosucrose synthesized by immobilized bi‐enzymes was significantly improved as compared to that synthesized by immobilized β‐fructofuranosidase. HPLC and NMR spectrum results confirmed the presence of lactosucrose during immobilized bi‐enzymes catalysis. Furthermore, a relatively high purity of lactosucrose was obtained (87.4% determined by HPLC) after separation with Diaion UBK535 calcium ester resin, and the optimal conditions for separation of lactosucrose were investigated. These results indicated that the co‐immobilization of β‐fructofuranosidase‐glucose oxidase was helpful to improve the production of lactosucrose with low costs, which can be used in continuous lactosucrose production in food industry in advantages of high stability and reusability. And the as‐prepared lactosucrose with high purity can be applied to many kinds of food as functional additives.

## 1 INTRODUCTION

Prebiotics are carbohydrate compounds that are nondigestible in the upper gastrointestinal tract but can be fermented by the intestinal microflora to selectively stimulate the growth of intestinal bacteria such as *Bifidobacteria* and *Lactobacilli* (Gibson, Probert, Loo, Rastall, & Roberfroid, 2004). Physiological benefits of prebiotics have been recognized as a consequence of the ability to maintain or restore a healthy gut microbiota (Silvério, Macedo, Teixeira, & Rodrigues, 2015). Lactosucrose has been proven to be an oligosaccharide functioning as prebiotic (Xu, Liu, Yu, Zhang, & Mu, 2018). It is reported that lactosucrose can promote the proliferation of Bifidobacterium in vivo better than other oligosaccharides (Arakawa et al., 2002). This probiotic effect promotes the maintenance of intestinal microflora and intestinal protection (Fujita, Ito, & Kishino, 2009). Moreover, lactosucrose can reduce body fat accumulation (Kimura, Nagata, Bryant, & Buddington, 2002), promote calcium absorption (Teramoto et al., 2006), and enhance amino acid metabolism (Ruan et al., 2013) and immune function (Arakawa et al., 2002).

Lactosucrose is a functional trisaccharide obtained through enzymatic synthesis. Lactose and sucrose, which are the most common and cheapest disaccharides exist in nature, are using as substrates for the production of lactosucrose. Sucrose is commonly used as sweetener in the food industry and has been confirmed to present risks for human health. Lactose is mainly produced from whey, which is discarded as wastes in food industry, resulting in environmental problems. Hence, the use of sucrose and lactose as substrates to synthesis prebiotics not only recovers the waste but also increases their added value. Lactosucrose is synthesized from sucrose and lactose under the catalysis of β‐fructofuranosidase (β‐d‐fructofuranoside fructohydrolase, EC 3.2.1.26) (Pilgrim et al., 2001). However, the production of lactosucrose is hampered by the costly use of β‐fructofuranosidase, which shows easy deactivation at higher temperature, product contamination and a low efficiency in transfructosylation activity (Smaali et al., 2012; Tonozuka et al., 2012). In addition, the glucose by‐product noncompetitively inhibits the transfructosylation activity of β‐fructofuranosidase, thereby causing low yields of lactosucrose (about 25%) and expensive product purification (Kawase, Pilgrim, Araki, & Hashimoto, 2001). Glucose oxidase can catalyze the conversion of glucose to gluconic acid, which eliminates the by‐product inhibition and greatly improves the conversion efficiency (Jong & Seung, 1993).

Enzyme immobilization is a common technology in industry as it enables the continuous use of enzymes, protects enzymes from denaturation, and can be easily recovered from the products (Singh, Saini, & Kennedy, 2010). In previous studies, various immobilization strategies have been introduced to immobilize β‐fructofuranosidase or glucose oxidase (Awad et al., 2013; Chen, Sheu, & Duan, 2014; Lorenzoni, Aydos, Klein, Rodrigues, & Hertz, 2014). However, the co‐immobilization of β‐fructofuranosidase and glucose oxidase for the production of lactosucrose is still seldom reported and lack of systematic study. Sol‐gel encapsulation possesses distinct physical and chemical characters compared with traditional immobilization methods due to its mild reaction conditions, wide applicability, and ability to maintain biomolecular structure and activity (Kauffmann & Mandelbaum, 1998). Moreover, it is reported that a sol–gel encapsulated enzyme in the hydrated pores can retain its solution structure and native function, thus remaining high activity retention (Smith et al., 2002).

Fueled by the widely use of lactosucrose in new functional foods as well as the advantageous aspect of immobilization in practical applications, studies were carried out to investigate the synthesis of lactosucrose using β‐fructofuranosidase and glucose oxidase co‐immobilized by sol–gel encapsulation, and the reaction conditions for production of lactosucrose using lactose and sucrose as subtracts were optimized. As a result, a stable immobilized bi‐enzymes system with high activity retention and an improved efficiency of lactosucrose conversion were obtained. The immobilized bi‐enzymes can be used for lactosucrose production in food industry with low cost.

## 2 MATERIALS AND METHODS

### 2.1 Materials

*Arthrobacter sp.10137* was purchased from the China Center of Industrial Culture Collection. β‐fructofuranosidase was obtained from *Arthrobacter sp.10137* and further purified in our laboratory (detailed at 2.2 and 2.3). The glucose oxidase was obtained from Novo Nordisk. Lactose (≥98%) and glucose (99%) were purchased from Sigma‐Aldrich Chemical Co. The standard of lactosucrose was purchased from Wako Pure Chemical Industries, Ltd. The other chemicals were of analytical grade and purchased from Sinopharm Chemical Reagent Co., Ltd.

### 2.2 Preparation of β‐fructofuranosidase

The preserved *Arthrobacter sp.10137* was first inoculated into activated medium, containing 2% (w/v) glucose, 0.15% (w/v) yeast extract, 0.01% (w/v) magnesium sulfate, 0.2% (w/v) monobasic potassium phosphate, and 0.6% (w/v) diammonium hydrogen phosphate (pH 7.0–7.2). After culturing at 30°C under gentle shaking for 24 hr, the activated strain was obtained. Then, 1 ml of the activated strain was inoculated into 100 ml of seed medium. The strain was cultured at 30°C under shaking with a speed of 125 r/min. After 24 hr of cultivation, 2 ml of the seed culture was inoculated into 100 ml of fermentation medium, containing 4% (w/v) sucrose, 1.2% (w/v) yeast extract, 0.8% (w/v) peptone, 0.2% (w/v) magnesium sulfate, 0.2% (w/v) potassium dihydrogen phosphate, and 0.4% diammonium hydrogen phosphate (pH 7.0–7.2). The strain was cultured at 30°C for 48 hr under shaking with a speed of 125 r/min. After that, the crude β‐fructofuranosidase was obtained through centrifugation for 20 min at 4000 r/min and then preserved at 4°C.

### 2.3 Enzyme purification

The crude β‐fructofuranosidase was further purified by ammonium sulfate precipitation. 30% of ammonium sulfate saturated solution was first used to remove impurities compounds; then, 70% saturated solution was added. The mixtures were centrifuged at 4000 r/min for 20 min, and the precipitates was collected and fully dissolved in phosphate buffer (50 mmol/L, pH 6.5). The purified enzyme was obtained after dialysis against the same phosphate buffer for 48 hr.

### 2.4 Co‐immobilization of β‐fructofuranosidase and glucose oxidase

Co‐immobilization of β‐fructofuranosidase and glucose oxidase was carried out according to a previously reported method (Jie Long et al., 2017). Firstly, 20 ml of purified β‐fructofuranosidase (1 mg/ml) and 20 ml of glucose oxidase (1 mg/ml) were added to the reaction kettle. Then, 10 ml of aqueous polyvinyl alcohol (PVA AH‐26) (4% w/v) and 10 ml of distilled water were added with 5 ml of aqueous sodium fluoride (NaF) (1 mol/L) as initiator under gentle shaking. Afterward, 7.4 ml of n‐octyltriethoxysilane (OTES) and 3.7 ml of tetraethoxysilane (TEOS) were added, and the mixtures were stirred at room temperature thoroughly overnight. The sol–gel immobilized bi‐enzymes were collected by centrifugation and washed three times with distilled water. The sol–gel matrix was controlled and prepared following the above steps without the addition of enzymes.

### 2.5 Activity assay of β‐fructofuranosidase

A reaction mixture of 0.5 ml β‐fructofuranosidase solution and 0.5 ml sucrose/lactose solution (pH 6.5, containing 20% (w/v) sucrose and 20% (w/v) lactose) was added to the tube and incubated in a water bath at 35°C for 8 hr. Then, the reactions were stopped by boiling for 10 min. One unit of enzyme activity was defined as the amount of enzyme required to produce 10^–9^ mol lactosucrose per minute at pH 6.5 and 35°C.

The concentration of lactosucrose was analyzed by an HPLC system equipped with a refractive index detector and an amino column (250mm × 4.6mm × 5mm, Thermo Fisher Scientific). The column was eluted with 90% (v/v) acetonitrile at a flow rate of 1 ml/min at 30°C.

The activity of β‐fructofuranosidase was calculated as follows:

(1) $$\text{Enzyme}\;\text{activity}\left(U\right)=\frac{c*10^{6}}{M*t*V}$$

where *c* is the content of lactosucrose (mg/ml), *M* is the molecular weight of lactosucrose (mg/mmol), t is the reaction time (min), and *V* is the enzyme volume (ml).

### 2.6 Activity assay of glucose oxidase

The activity of glucose oxidase was measured according to the DNSA method (Wang, Zhu, & Zhou, 2011). 1 ml of β‐d‐glucose solution (1 mg/ml), 0.1 g glucose oxidase (28,000 U/g), and 3 ml of acetic buffer (pH 6.0) were added and incubated at 30°C for 30 min under a gentle shaking. The reaction was stopped by boiling for 10 min. Then, 2 ml of DNSA was added and the mixture was boiling for 5 min. After cooled to room temperature, the mixture was diluted to 25 ml and quantified by measuring absorbance at 520 nm using a spectrophotometer (TU1900, Persee Ltd.). A unit of glucose oxidase activity is defined as the amount of enzyme required to oxidize 1.0 μmol of β‐d‐glucose to gluconic and hydrogen peroxide per minute at 30°C.

### 2.7 Total activity assay of bi‐enzymes

0.5 ml β‐fructofuranosidase (1 mg/ml), 0.5 ml glucose oxidase (1 mg/ml), and 0.5 ml sucrose/lactose solution [pH 6.5, containing 20% (w/v) sucrose and 20% (w/v) lactose] were added and incubated in a water bath at 35°C for 8 hr. The reaction was stopped by boiling for 10 min. One unit of enzyme activity was defined as the amount of enzyme required to produce 10^–9^ mol lactosucrose per minute at pH 6.5 and 35°C.

The concentration of lactosucrose was analyzed by an HPLC system according to the method detailed at 2.5.

### 2.8 Assay of amount of immobilized protein, activity retention, and loading efficiency

After co‐immobilization, the amount of protein in the supernatant was determined according to the Bradford protein assay method (Franzreb, Siemann‐Herzberg, Hobley, & Thomas, 2006). The amount of protein immobilized was calculated by the difference between the amounts of protein in the supernatant and the initial protein added.

The activity retention and enzyme loading efficiency were calculated according to the following equations:

(2) $$\text{Activity}\;\text{retention}=\frac{A_{\text{im}}}{A_{0}-A_{i}}*100\%$$

where *A*_0_ is the total specific activity of bi‐enzymes, *A*_i_ is the total specific activity of the supernatant solution, and *A*_im_ is the total specific activity of immobilized bi‐enzymes.

(3) $$\text{Enzyme}\;\text{loading}\;\text{efficiency}=\frac{C_{0}V_{0}-C_{i}V_{i}}{C_{0}V_{0}}\times 100\%$$

where *C*_0_ is the initial protein concentration (mg/ml), *V*_0_ is the initial volume (mL) of the free β‐fructofuranosidase and glucose oxidase solution added for co‐immobilization, *C*_i_ is the total protein concentration (mg/ml) of the supernatant, and *V*_i_ is the total volume (ml) of the supernatant after co‐immobilization.

### 2.9 Reusable stability

The reusable stability was assayed by incubating immobilized bi‐enzymes with sucrose/lactose solution (containing 20% (w/v) sucrose and 20% (w/v) lactose) in phosphate buffer (50 mmol/L, pH 6.5) in a water bath of 35°C for 8 hr. The reaction mixture was then centrifuged at 10,000 r/min for 10 min to collect the residual immobilized bi‐enzymes. The fresh sucrose/lactose solution [containing 20% (w/v) sucrose and 20% (w/v) lactose] was added to the residual immobilized bi‐enzymes for the next cycle of reaction. The above steps were repeated fifteen times. The activity of the first run was defined as 100%.

### 2.10 Optimization of synthesis conditions of lactosucrose

#### 2.10.1 Sucrose‐to‐lactose ratio

Sucrose and lactose were used as substrates; the total substrate concentration was kept at 40%. 0.5 ml of substrate solution (sucrose‐to‐lactose ratio was 3:1, 2:1, 1:1, 1:2, 1:3 w/w, respectively) and 1 g of immobilized bi‐enzymes (1.2 mg/g·sol) were added into the tube and then incubated in a water bath at 35°C for 8 hr (pH 6.5).

#### 2.10.2 Substrate concentration

The sucrose‐to‐lactose ratio was kept at 1:1 (w/w). 0.5 ml of substrate solution (the concentration was 10%, 15%, 20%, 25%, respectively) and 1 g of immobilized bi‐enzymes (1.2 mg/g·sol) were added into the tube and then incubated in a water bath at 35°C for 8 hr (pH 6.5).

#### 2.10.3 Treatment times

The total substrate concentration and sucrose‐to‐lactose ratio were kept at 20% and 1:1 (w/w), respectively. 0.5 ml of substrate solution and 1 g of immobilized bi‐enzymes (1.2 mg/g·sol) were added into the tube and then incubated in a water bath at 35°C for 2 hr, 4 hr, 6 hr, 8 hr, 10 hr or 12 hr (pH 6.5).

#### 2.10.4 pH value

The total substrate concentration and sucrose‐to‐lactose ratio were kept at 20% and 1:1 (w/w), respectively. 0.5 ml of substrate solution and 1 g of immobilized bi‐enzymes (1.2 mg/g·sol) were added into the tube and then incubated in a water bath at 35°C for 8 hr. The pH value was set at 4.0, 5.0, 6.0, 6.5, 7.0, 8.0, or 9.0.

#### 2.10.5 Temperature

The total substrate concentration and sucrose‐to‐lactose ratio were kept at 20% and 1:1 (w/w), respectively. 0.5 ml of substrate solution and 1 g of immobilized bi‐enzymes (1.2 mg/g·sol) were added into the tube and then incubated in a water bath at 30, 35, 40, 45, 50, or 55°C for 8 hr (pH 6.5).

#### 2.10.6 Enzyme concentration

The total substrate concentration and sucrose‐to‐lactose ratio were kept at 20% and 1:1 (w/w), respectively. 0.5 ml of substrate solution and 1 g of immobilized bi‐enzymes (enzyme concentration was 0.5, 1.0, 1.5, 2.0 mg/g·sol, respectively) were added into the tube and then incubated in a water bath at 40°C for 8 hr (pH 6.5).

#### 2.10.7 β‐fructofuranosidase‐to‐glucose oxidase ratio

In the process of immobilization, the β‐fructofuranosidase‐to‐glucose oxidase ratio was set as 5:1, 4:1, 3:1, 2:1, 1:1, 1:2, or 1:3 (w/w), respectively; then, the as‐prepared different immobilized bi‐enzymes were used to synthesize lactosucose. The total substrate concentration and sucrose‐to‐lactose ratio were kept at 20% and 1:1 (w/w), respectively. 0.5 ml of substrate solution and 1 g of immobilized bi‐enzymes (1.0 mg/g·sol) were added into the tube and then incubated in a water bath at 40°C for 8 hr (pH 6.5).

### 2.11 Purification of lactosucrose

#### 2.11.1 Effect of different resins on separation of lactosucrose

The effect of two calcium‐type resins, Diaion UBK535 and UBK555, on the separation of lactosucrose was studied. The resin was first soaked in distilled water for 3 hr and then poured into the chromatography column (70 × 1 cm) with a thermal insulation jacket. After the resin was filled, a water layer of 2 cm high was left on the surface. The ion exchange resin column was made thermostat at 50°C by the heating water flowing through the insulation jacket. One millilitre of sample was applied to the resin and eluted with distilled water at a flow rate of 0.3 ml/min. The eluent was analyzed by an HPLC system.

#### 2.11.2 Effect of elution velocity, loading amount, and temperature on separation of lactosucrose

To study the effect of elution velocity on the separation of lactosucrose, 1 ml of sample was applied to the ion exchange resin column and eluted with distilled water at a flow rate of 0.1 ml/min, 0.3 ml/min, or 0.5 ml/min. The column was made thermostat at 50°C.

To study the effect of loading amount on the separation of lactosucrose, 1 ml, 2 ml, or 3 ml of sample was applied to the ion exchange resin column and eluted with distilled water at a flow rate of 0.3 ml/min. The column was made thermostat at 50°C.

To study the effect of temperature on the separation of lactosucrose, 1 ml of sample was applied to the ion exchange resin column and eluted with distilled water at a flow rate of 0.3 ml/min. The column was made thermostat at 40, 50, 60, or 70°C.

### 2.12 Yield and purity of lactosucrose

The yield of lactosucrose was calculated according to the following equation:

(4) $$\text{Yield}=\frac{m_{1}}{m_{0}}\;\times \;100\%$$

where $m_{1}$ is the amount of lactosucrose eluted (mg); $m_{0}$ is the amount of lactosucrose in the sample (mg).

(5) $$\text{Purity}=\frac{m_{i}^{\prime }}{m_{i}}\;\times \;100\%$$

where $m_{i}^{{}^{\prime }}$ is the amount of lactosucrose eluted at a certain time (mg); $m_{i}$ is the total amount of carbohydrate eluted at a certain time (mg).

### 2.13 NHR analysis

The product purified by resin was collected and concentrated by rotary evaporation and lyophilized. The lyophilized powder was reconstituted with D_2_O and characterized by ^13^C NMR and ^1^H NMR.

### 2.14 Statistical analysis

Experiments were triplicated, and the results are presented as mean ± standard deviation (*SD*). Significant differences were determined statistically using one‐way analysis of variance (ANOVA) post hoc Tukey test and accepted at *p* < .05.

## 3 RESULTS AND DISCUSSION

### 3.1 Co‐immobilization of β‐fructofuranosidase and glucose oxidase

**Figure 1:**
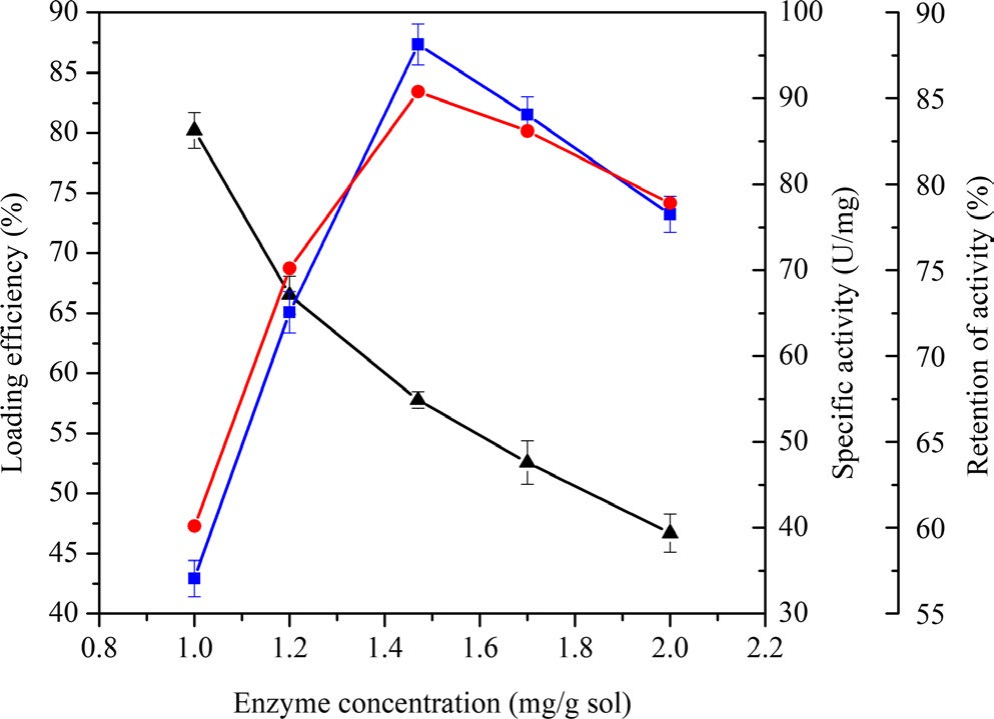
Effect of enzyme concentration on enzyme loading efficiency (▲), specific activity (), and activity retention () of immobilized bi‐enzymes. Experiments were triplicated, and the results are presented as mean ± standard deviation (*SD*)

In the process of co‐immobilizing β‐fructofuranosidase and glucose oxidase by sol–gel encapsulation, the extent of immobilization was greatly affected by variations in enzyme concentration. The effect of enzyme concentration on the enzyme loading efficiency, specific activity, and activity retention of immobilized bi‐enzymes is shown in Figure 1. The enzyme loading efficiency decreased gradually with the increase of enzyme concentration in a range of 1.11–2.01 mg/g·sol, while the specific activity and activity retention both increased first and then decreased, and the maximum values (96.3 U/mg and 85.4%, respectively) were obtained at an enzyme concentration of 1.47 mg/g·sol, indicating that the sol–gel matrix was not saturated with enzymes. At higher concentrations, the reduced activity of enzyme was due to the aggregation or the formation of a multi‐layer of enzyme, which increases the mass transfer resistance.

### 3.2 Operational stability of the immobilized bi‐enzymes

**Figure 2:**
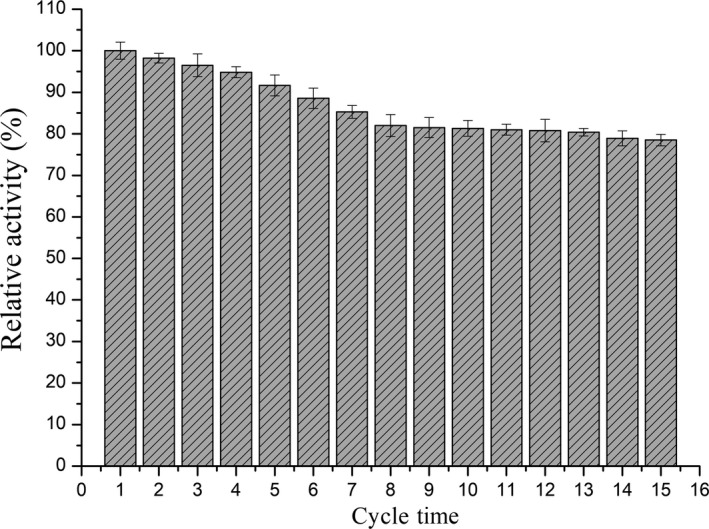
Reusability of immobilized bi‐enzymes. Experiments were triplicated, and the results are presented as mean ± standard deviation (*SD*)

Operational stability of the immobilized enzyme was highly related to the practical applications, and the immobilized enzyme with improved operational stability is more favorable to the practical application compared to free enzymes (Long et al., 2015). To investigate the operational stability of the immobilized bi‐enzymes, fifteen consecutive reuse cycles were conducted under the investigated optimal conditions. The activity of the first run was defined as 100%. As shown in Figure 2, the immobilized bi‐enzymes maintained the activity well during the consecutive reuse cycles. After 10 and 15 consecutive reuses, the immobilized bi‐enzymes retained more than 81.3% and 78.5% of its initial activity, indicating that the immobilized bi‐enzymes had a good operational stability and showed great potential in the continuous production of lactosucrose in food industry.

### 3.3 Effect of substrate ratio and concentration on lactosucrose production

**Figure 3:**
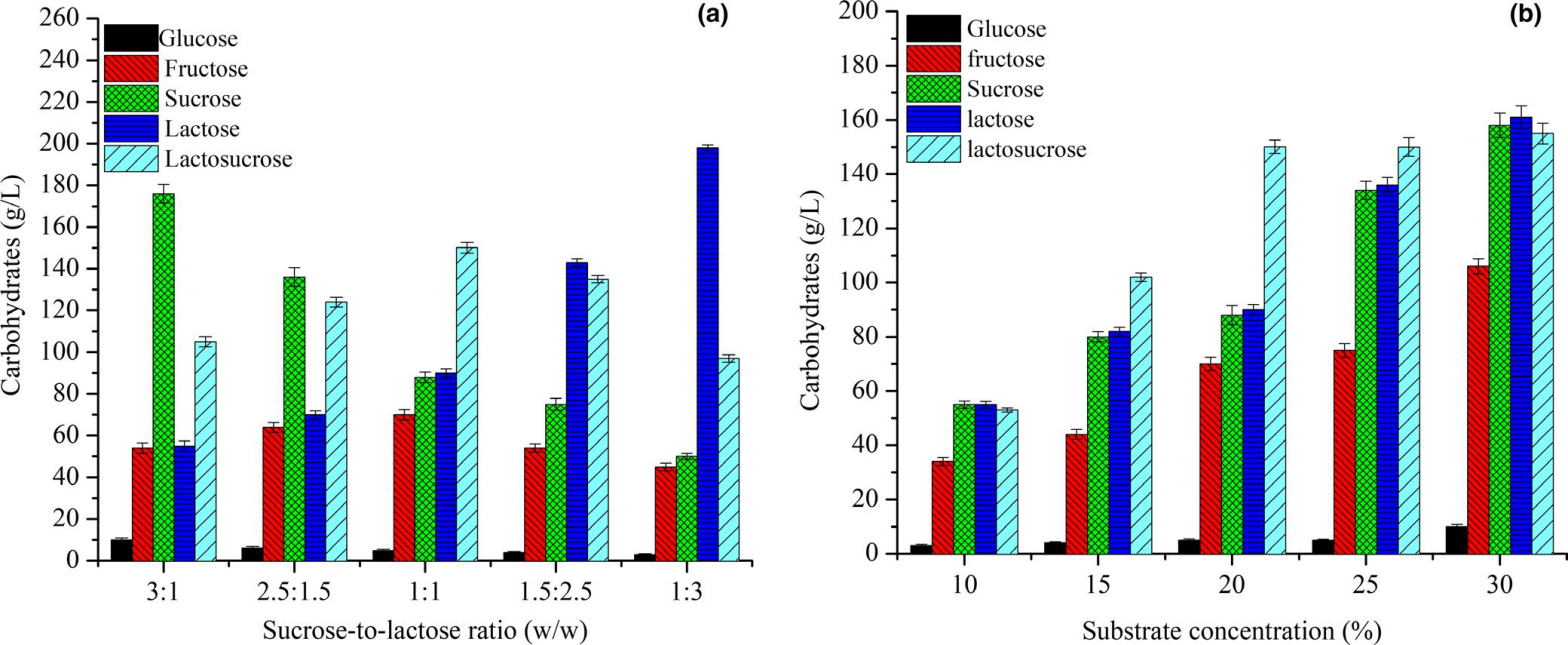
Effect of substrate ratio (a) and substrate concentration (b) on the synthesis of lactosucrose. Experiments were triplicated, and the results are presented as mean ± standard deviation (*SD*)

The synthesis of lactosucrose by β‐fructofuranosidase belongs to the fructosyl transfer reaction, involving two substrates (sucrose and lactose); therefore, the ratio of these two substrates in the reaction system greatly impacts the synthesis of lactosucrose. The effect of sucrose‐to‐lactose ratio on the yield of lactosucrose synthesized by immobilized bi‐enzymes is shown in Figure 3a. As the substrate ratio changes, the glucose content in the product was always less than 10 g/L, indicating the good control of glucose by‐product due to the quick catalysis of glucose into gluconic acid by glucose oxidase. The highest content of lactosucrose (150.1 g/L) was obtained at the sucrose‐to‐lactose ratio of 1:1. Li et al. (2015) reported the similar results for the synthesis of lactocucrose by the purified recombinant levansucrase from *leuconostoc mesenteroides* B‑512 FMC.

The effect of substrates concentration on the lactosurose production is shown in Figure 3b. The contents of lactosucrose and fructose increased slowly with the increase of substrate concentration, and the maximum yield of lactosucrose (150.8 g/L) was obtained at the substrates concentration of 20%. With the further increase of substrate concentration, the content of lactosucrose tended to be stable, indicating that the transfructose‐based reaction by immobilized bi‐enzymes reached saturation, and the increase in fructose content may be due to the hydrolysis of sucrose.

### 3.4 Effect of treatment times, pH value, and temperature on lactosucrose production

**Figure 4:**
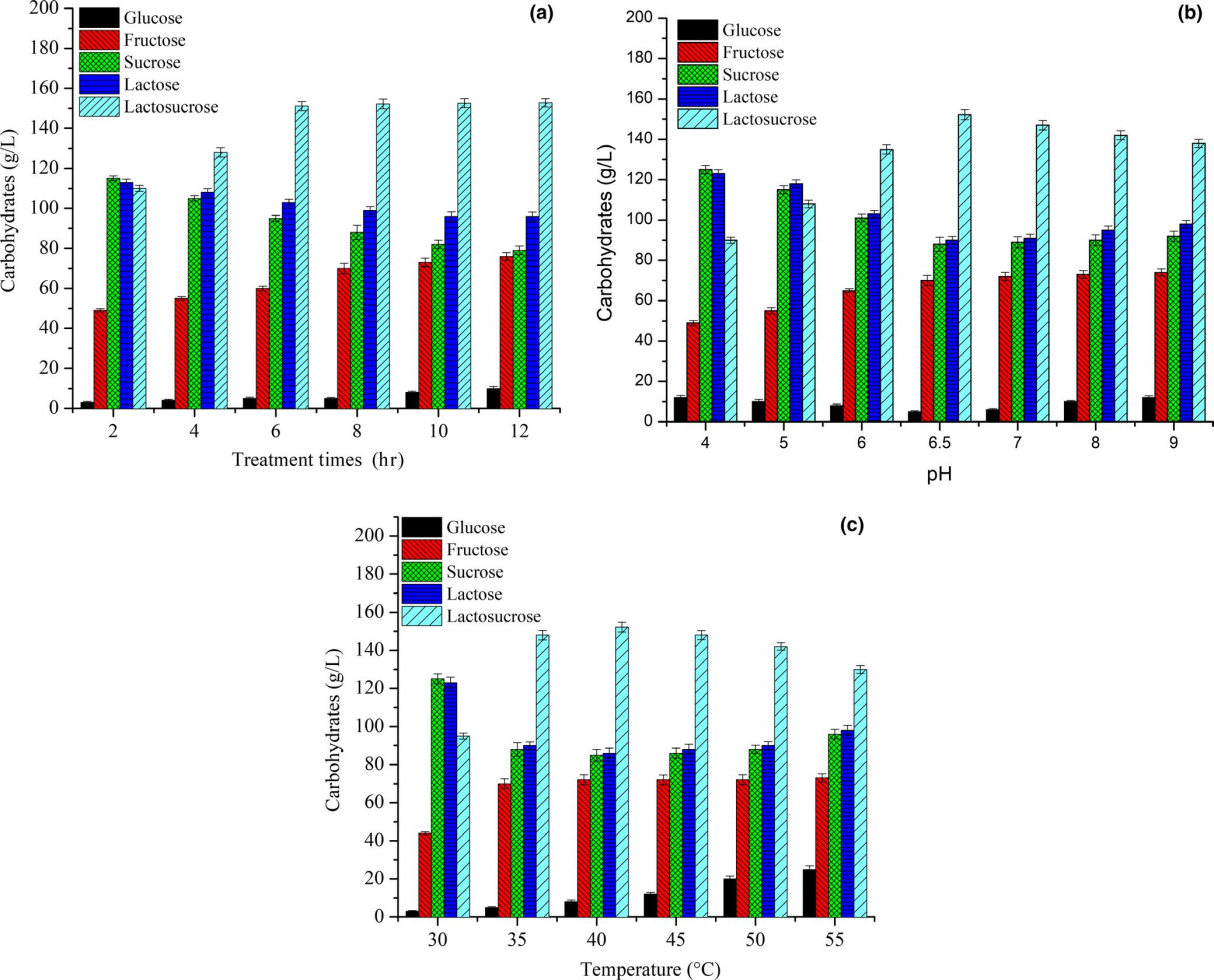
Effect of treatment times (a), pH (b), and temperature (c) on the synthesis of lactosucrose. Experiments were triplicated, and the results are presented as mean ± standard deviation (*SD*)

The effect of treatment times on the lactosucrose production is shown in Figure 4a. Over the first 8 hr lactosurose synthesized improved significantly, reaching a maximum content of 152.2 g/L for a treatment time of 8 hr. After 8 hr, lactosucrose yield did not significantly increase, and the fructose content continued to increase, indicating that the fructosyl transfer reaction gradually reached saturation, and lactosucrose began to self‐hydrolyze; therefore, excessive treatment time was not conducive to the synthesis of lactosucrose.

The optimal pH for lactosucrose production was investigated with the pH variation from 4.0 to 9.0. As shown in Figure 4b, the content of lactosucrose gradually increased with the increased pH value, and the acidic condition exhibited a great influence on the synthesis of lactosucrose by immobilized bi‐enzymes; especially at pH 4.0, the yield of lactosucrose was only 90.2 g/L. The highest yield of lactosucrose (152.2 g/L) was obtained at pH 6.5. With the further increase in pH, the content of the synthesized lactosucrose slowly decreased (140.2 g/L at pH 9.0).

Temperature is an important factor for industrial production of lactosucrose. Increased temperature is helpful for increasing the substrate solubility and improving transfructosylation rate (Choi, Kim, Kim, Jung, & Oh, 2004). The influence of temperature on the lactosucrose production was also studied. As shown in Figure 4c, the yield of lactosucrose reached the highest value (153.1 g/L) at 40°C. With increased temperatures between 35 and 45°C, the synthesized lactosucrose content was relatively high and stable; however, when the temperature went above 45°C, the yield of lactosucrose gradually decreased, might due to the changes in structure of the immobilized bi‐enzymes with elevated temperature as well as the accumulation of the glucose by‐product. The results also indicated that immobilization greatly improved the thermal stability of β‐fructofuranosidase as compared to free β‐fructofuranosidase, by shifting the optimum temperature from 35 to 40°C (data were not shown). These results suggested that immobilization was favorable to the fructosyl transfer reaction of β‐fructofuranosidase during heat treatment with high temperature, which is helpful for improving transfructosylation rate.

### 3.5 Effect of enzyme concentration and β‐fructofuranosidase–to‐glucose oxidase ratio on lactosucrose production

**Figure 5:**
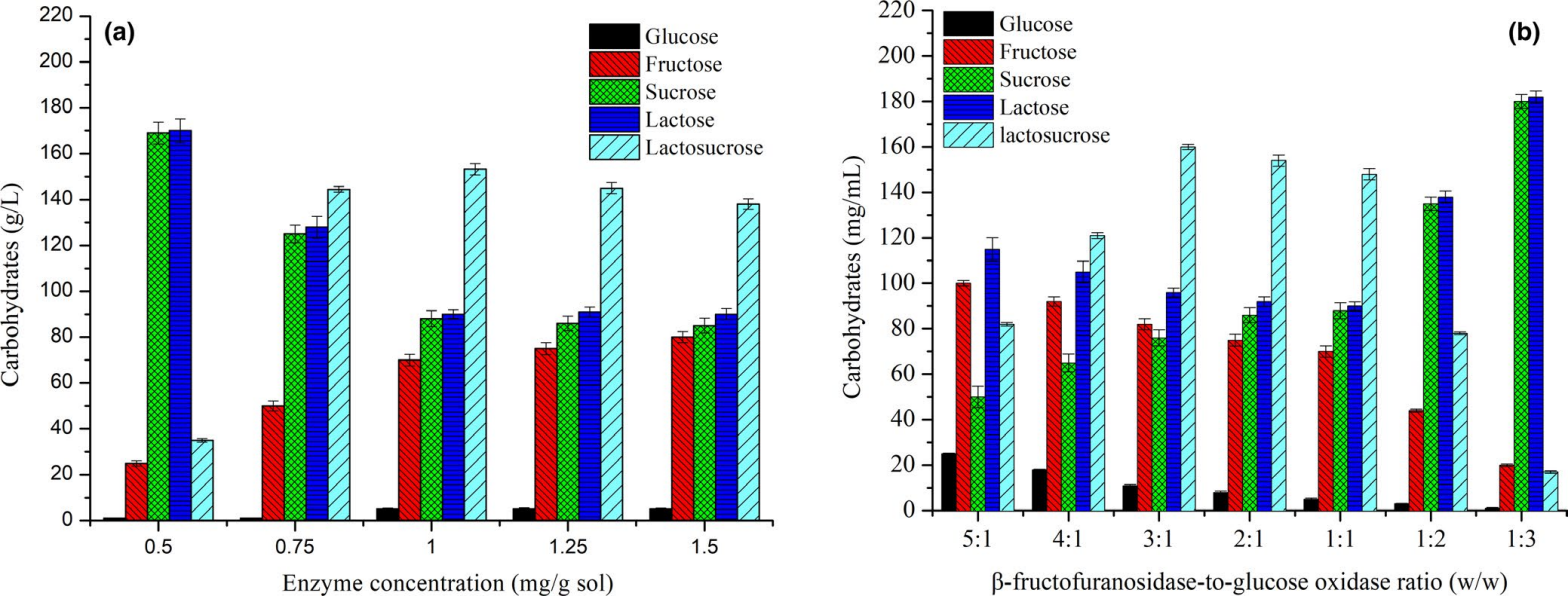
Effect of enzyme concentration (a) and β‐fructofuranosidase‐to‐glucose oxidase ratio (b) on the synthesis of lactosucrose. Experiments were triplicated, and the results are presented as mean ± standard deviation (*SD*)

Different enzyme concentrations from 0.5 to 1.5 mg/g·sol were introduced to investigate the synthesis of lactosucrose, and the results are presented in Figure 5a. The content of lactosucrose synthesized increased significantly (*p* < .01) when increasing the enzyme concentration from 0.5 to 1.0 mg/g·sol and reached the highest value of 153.3 g/L. As the enzyme concentration increased from 1.0 to 1.5 mg/g·sol, the excessive immobilized bi‐enzymes began to hydrolyze the lactosucrose, resulting in a slight decrease in lactosucrose production and increase in lactose and fructose content.

Because each enzyme in co‐immobilized system had its own catalytic property, and their synergistic effect on the synthesis of lactosucrose depended on the ratio of β‐fructofuranosidase‐to‐glucose oxidase. Based on the above results, the optimal conditions for lactosucrose synthesis by immobilized bi‐enzymes were obtained, in which the substrate ratio and concentration, treatment times, pH, temperature, enzyme concentration were 1:1 (w/w), 20% (W/V), 8 hr, pH 6.5, 40°C, and 1.0 mg/g·sol, respectively. Under the optimal conditions, the effect of β‐fructofuranosidase‐to‐glucose oxidase ratio on lactosucrose production was investigated and the results are shown in Figure 5b. As the ratios of β‐fructofuranosidase‐to‐glucose oxidase varied from 5:1 to 3:1, the content of lactosucrose gradually increased and reached the highest value as 160.8 g/L, suggesting that the decrease in β‐fructofuranosidase lowered the further hydrolysis of lactosucrose into lactose and fructose, as confirmed by the decrease in contents of lactose and fructose. The further decrease in β‐fructofuranosidase content (from 3:1 to 1:3) limited the production of lactosucrose, due to the existing β‐fructofuranosidase cannot fully catalyzed sucrose and lactose.

Moreover, the yield of lactosucrose synthesized by co‐immobilized β‐fructofuranosidase‐glucose oxidase was significantly improved as compared to that synthesized by immobilized β‐fructofuranosidase (100.8 g/L), indicating that the addition of glucose oxidase realized the oxidation of the by‐product glucose to gluconic acid and eliminated the substrate inhibition.

### 3.6 Comparison of the yield of lactosucrose

The yield of lactosucrose is affected by factors such as the type of enzymes, the source of microorganisms, and the form of enzymes. In recent years, β‐fructofuranosidase, levansucrase, and β‐galactosidase are mostly enzymes used in the synthesis of lactosucrose. The comparison between our results and previous reported results is presented in Table 1. For lactosucrose synthesized by β‐fructofuranosidase, Ikegaki & Park (1997) founded that the content of lactosucrose synthesized by crude β‐fructofuranosidase from *Bacillus sp. 417* was 54 g/L. Mikuni et al. (2000) reported the synthesis of lactosucrose by immobilized β‐fructofuranosidase from *Arthrobacter sp. K‐1*, and the corresponding content was 120 g/L, which is lower than our result (160.8 g/L). For lactosucrose synthesized by levansucrase, the emphasis was focused on the use of genetic modified levansucrase. It seems that different genetic modification methods had different effects on the production of lactosucrose. Li et al. (2015) used a recombinant levansucrase from *Leuconostoc mesenteroides B‐512 FMC* to synthesize lactosucrose, and the corresponding content was 224 mg/ml. However, Lu et al. (2014) also employed a recombinant levansucrase (from *Bacillus licheniformis 8‐37‐0‐1*) to synthesize lactosucrose, and the corresponding content was only 88 mg/ml. Xu et al. (2018) reported the synthesis of lactosucrose using a recombinant levansucrase from *Brenneria goodwinii* and obtained the similar results (100 g/L). Moreover, the highest yield of lactosucrose (285 g/L) was reported by Han et al. (2007) using levansucrase from *Pseudomonas aurantiaca*, but the substrate concentration was very high (510 g/L sucrose and 360 g/L lactose). For lactosucrose synthesized by β‐galactosidase, the yield of lactosucrose was relatively low, for example, the content of lactosucrose synthesized by β‐galactosidase derived from *Bacillus circulans* was only 56 g/L (Li et al., 2009). Overall, our research fulfilled the improvement of yield of lactosucrose, and the immobilized bi‐enzymes showed great industrial application potential in lactosucrose production in advantages of high stability and reusability.

**Table 1:** Lactosucrose production by enzymes from various microorganisms

Enzyme	Microorganism		Optimal temperature (°C)	Substrates concentration (g/L) (sucrose + lactose)	Yield (g/L)
β‐fructofuranosidase	*Arthrobacter sp. 10137* (current work)	Immobilized bi‐enzymes	40	200 + 200	160.8
	*Arthrobacter sp. K‐1* (Fujita, Kara, Hashimoto, & Kitahata, 1990)	Crude enzyme	55	200 + 200	135
	*Arthrobacter sp. K‐1* (Mikuni et al., 2000)	Immobilized enzyme	55	200 + 200	120
	*Bacillus *sp.* 417* (Ikegaki & Park, 1997)	Crude enzyme	45	100 + 100	54
Levansucrase	*Leuconosto mesenteroides B‐512 FMC* (Li et al., 2015)	Purified recombinant enzyme	50	270 + 270	224
	*Bacillus licheniformis 8‐37‐0‐1* (Lu et al., 2014)	Purified recombinant enzyme	40	171 + 171	88
	*Bacillus natto* (Takahama et al., 1991)	Purified enzyme	35	85.5 + 85.5	53
	*Brenneria goodwinii* (Xu et al., 2018)	Purified recombinant enzyme	35	180 + 180	100
	*Pseudomonas aurantiaca* (Han et al., 2007)	Crude enzyme	45	510 + 365	285
	*Zymomonas mobilis* (Yanase et al., 1992)	Crude enzyme	23	180 + 180	103
	*Sterigmatomyces elviae mutant* (Han et al., 2007)	Immobilized mutant cell	50	250 + 250	192
	*Bacillus methylotrophicus SK 21.002 *(Wu, Zhang, Mu, Miao, & Jiang, 2015)	Crude enzyme	37	200 + 200	143
β‐galactosidase	*Bacillus circulans* (Li et al., 2009)	Crude enzyme	40	300 + 300	56

Note

Experiments were triplicated, and the results are presented as mean ± standard deviation (*SD*).

### 3.7 Purification of lactosucrose

**Figure 6:**
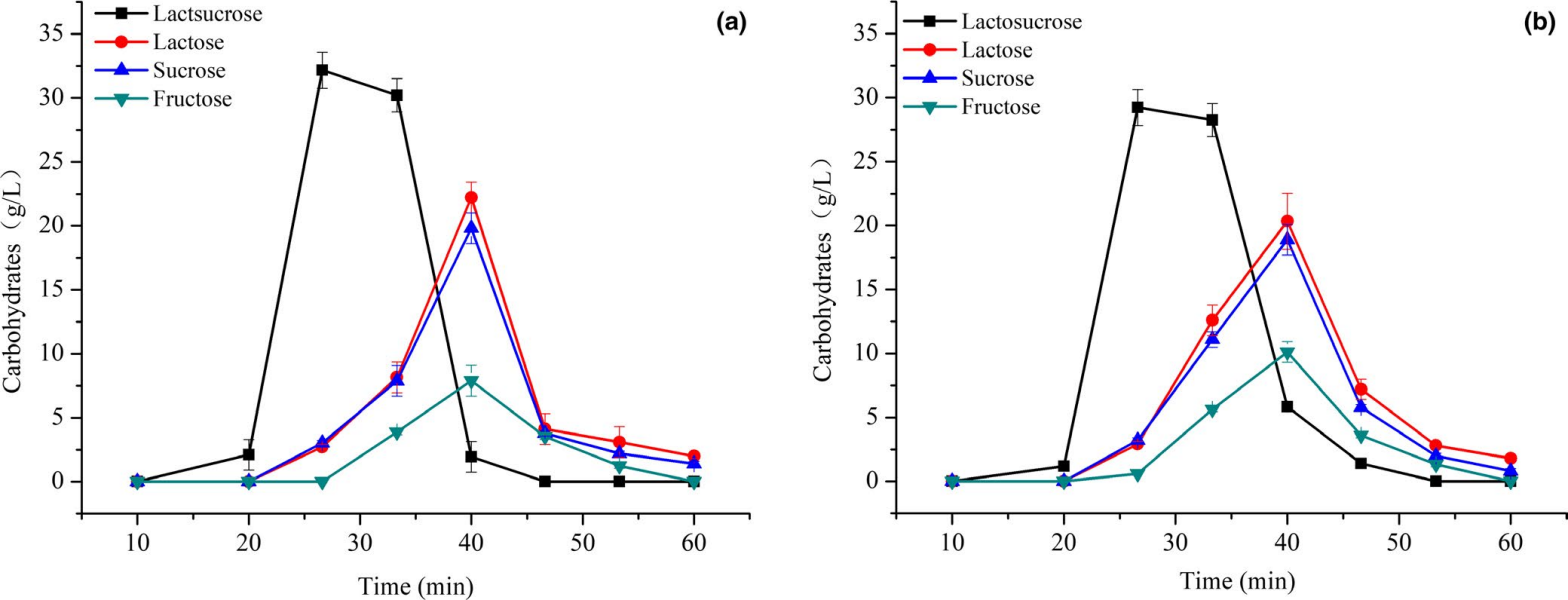
Elution curves of lactosucrose using UBK535 calcium ester resins (a) and UBK555 calcium ester resins (b). Experiments were triplicated, and the results are presented as mean ± standard deviation (*SD*)

Although we had obtained the high production of lactosucrose using immobilized bi‐enzymes system, there were still other undesired carbohydrates required to be removed to improve the purity of target lactosucrose. Chromatographic separation has been widely used in medicine, food, and fermentation industries. The lactosucrose synthesized by immobilized bi‐enzymes were further purified by Diaion UBK535 and UBK555 calcium ester resins, and the results are shown in Figure 6 and Table 2. No significant difference (*p* > .05) in adsorption capacity was observed between both resins. The lactosucrose can be completely separated from the other three carbohydrates, the elution of which just reached maximum when the lactosucrose was eluted completely. The low content of gluconic acid in products may be due to its strong adsorption capacity to the resin. However, UBK535 calcium ester resins exhibited better separation capacity than UBK555 in the aspects of resolution, yield and purity (Table 2). Therefore, UBK535 calcium ester resins were used in the subsequent studies.

**Table 2:** Effect of different resins on the separation efficiency of lactosucrose

Type	Resolution	Yield (%)	Purity (%)
UBK535	0.50	88.6 ± 0.2	84.9 ± 0.2
UBK555	0.45	84.9 ± 0.1	81.2 ± 0.1

Note

Experiments were triplicated, and the results are presented as mean ± standard deviation (*SD*).

**Figure 7:**
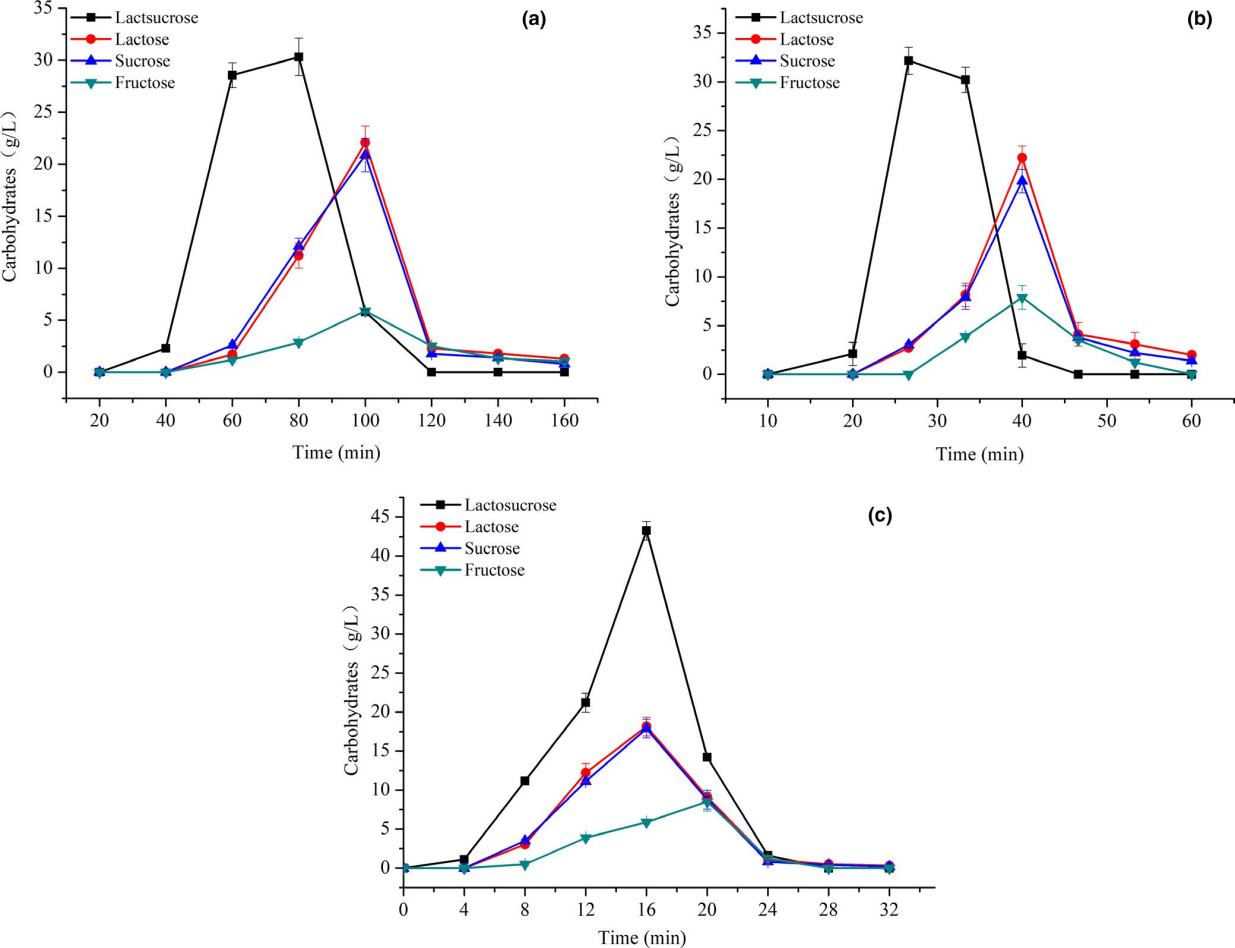
Elution curves of lactosucrose at a flow rate of 0.1 ml/min (a), 0.3 ml/min (b), and 0.5 ml/min (c). Experiments were triplicated, and the results are presented as mean ± standard deviation (*SD*)

Elution flow rate is one of the key factors affecting the separation of lactosucrose. Effect of elution velocity on separation of lactosucrose is presented in Figure 7 and Table 3. The lactosucrose can be quickly (just 46 min) separated from the other three carbohydrates at a flow rate of 0.3 ml/min. The separation efficiency reduced at a flow rate of 0.1 ml/min, with 2 hr elution of lactosucrose. Moreover, the resolution and purity of lactosucrose eluted at a flow rate of 0.3 ml/min were both higher than those eluted at a flow rate of 0.1 ml/min. However, the lactosucrose cannot be separated from the other three carbohydrates at a flow rate of 0.5 ml/min.

**Table 3:** Effect of elution velocity on the separation efficiency of lactosucrose

Flow rate (ml/min)	Resolution	Yield (%)	Purity (%)
0.1	0.44	89.3 ± 0.2	83.7 ± 0.1
0.3	0.50	88.6 ± 0.2	84.9 ± 0.2
0.5	0	97.4 ± 0.12	50.7 ± 0.3

Note

Experiments were triplicated, and the results are presented as mean ± standard deviation (*SD*).

**Figure 8:**
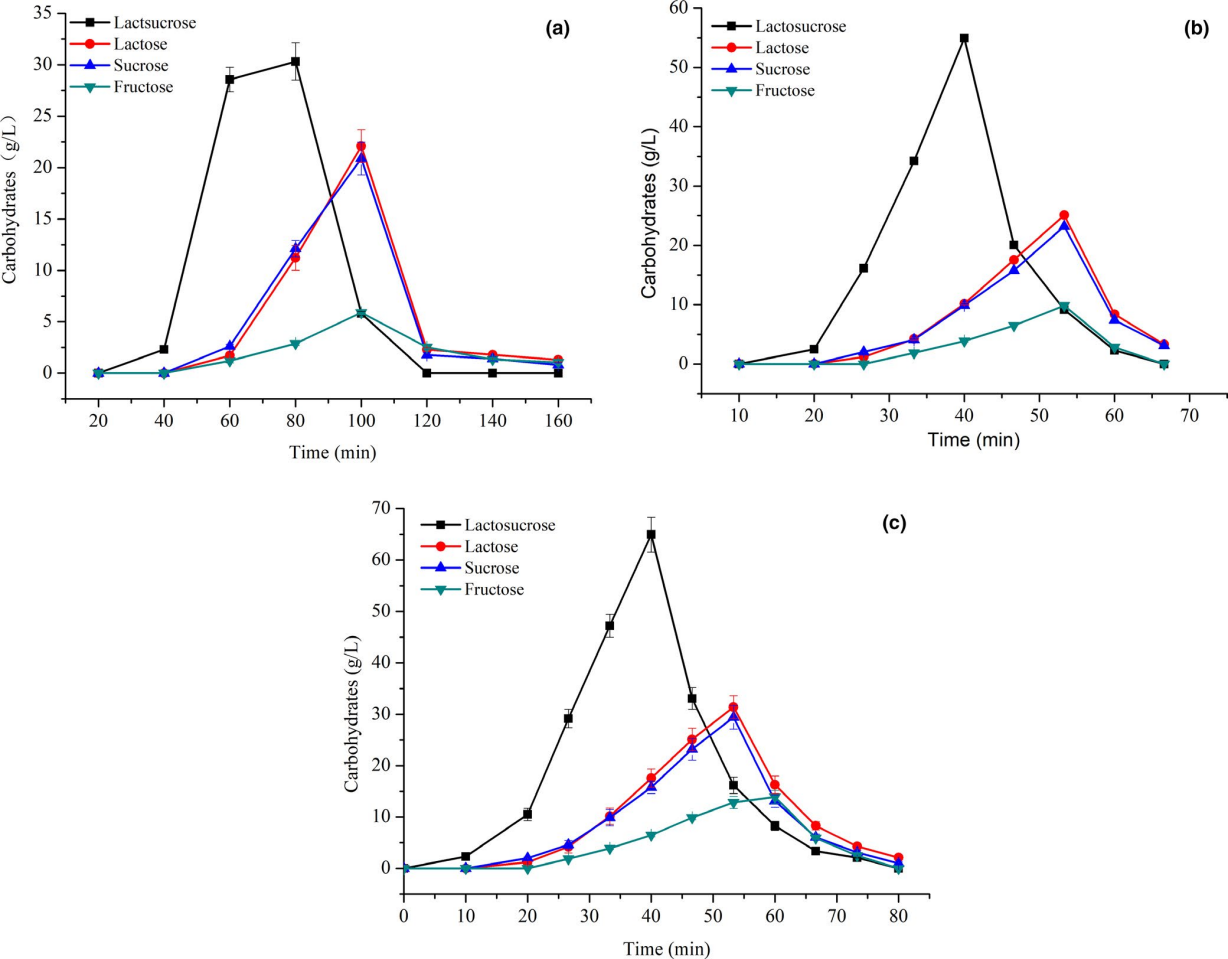
Elution curves of lactosucrose under loading amount of 1 ml (a), 2 ml (b), or 5 ml (c). Experiments were triplicated, and the results are presented as mean ± standard deviation (*SD*)

An increased loading amount can improve the productivity of chromatography column, but the separation efficiency will be decreased due to the saturated loading. Therefore, separation of lactosucrose variation in loading amount was investigated, and the results are presented in Figure 8 and Table 4. The adsorption capacity of the resin approached saturation at the loading amount of 1 ml, and the elution time of the lactosucrose gradually increased with the increase of loading amount. The highest yield of lactosucrose was obtained at the loading amount of 2 ml, but the corresponding resolution and purity were relatively low. The resolution and purity of lactosucrose reached the maximum values at the loading amount of 1.0 ml. Based on the above results, the optimal loading amount for the separation of lactosucrose was 1 ml.

**Table 4:** Effect of loading amount on the separation efficiency of lactosucrose

Loading amount (ml)	Resolution	Yield (%)	Purity (%)
1	0.50	88.6 ± 0.2	84.9 ± 0.2
2	0.33	92.9 ± 0.3	68.0 ± 0.1
5	0.21	57.9 ± 0.2	61.9 ± 0.4

Note

Experiments were triplicated, and the results are presented as mean ± standard deviation (*SD*).

**Figure 9:**
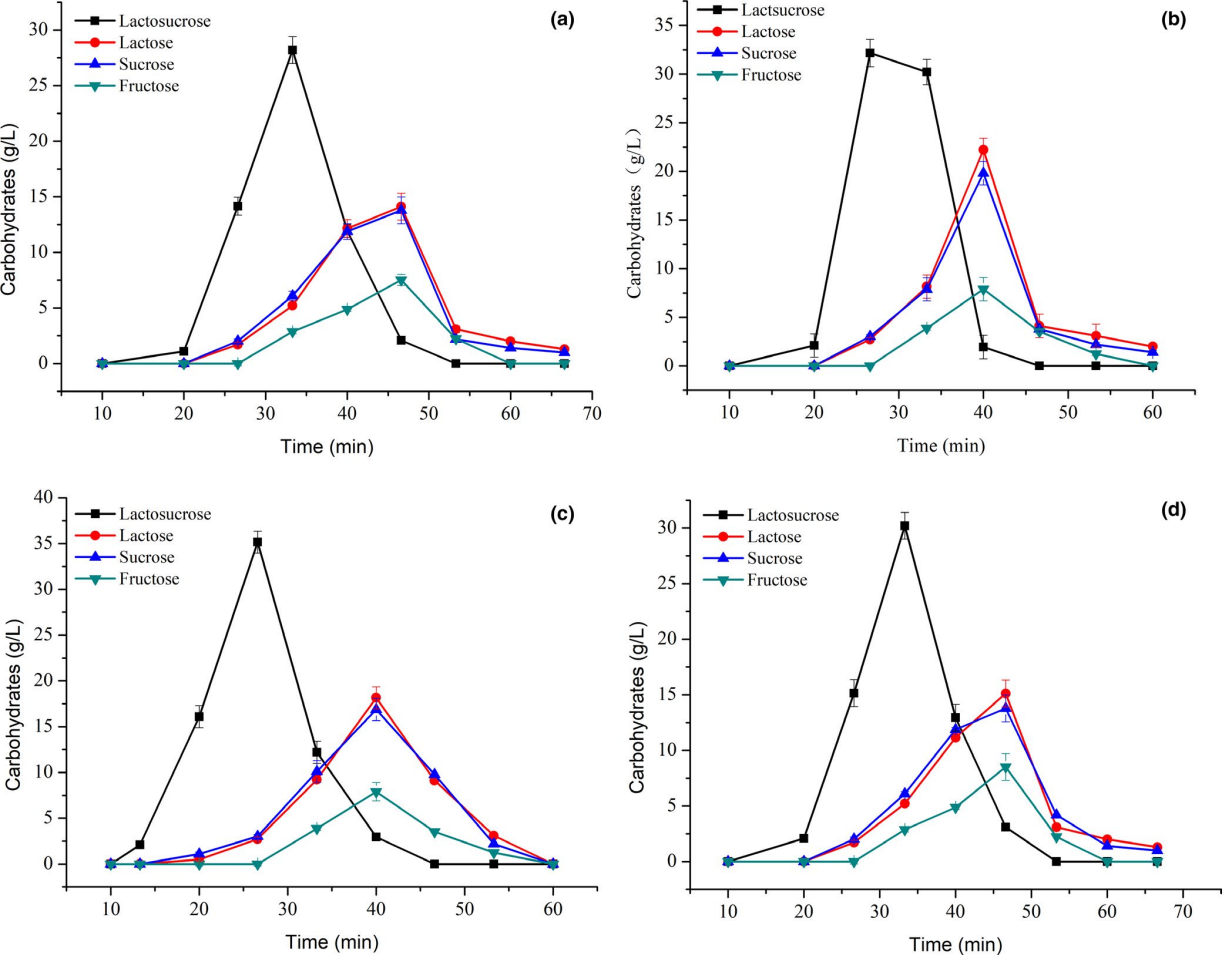
Elution curve of lactosucrose under different temperatures. The different temperatures were: (a) 40°C; (b) 50°C; (c) 60°C; (d) 70°C. Experiments were triplicated, and the results are presented as mean ± standard deviation (*SD*)

The separation of lactosucrose was also affected by temperature in two aspects: the viscosity of the lactosucrose and the mass transfer between carbohydrates and resin. The effect of temperature on the separation of lactosucrose is shown in Figure 9 and Table 5. As temperature increased from 40 to 60°C, the time for elution of lactosucrose was gradually shortened, and the resolution, yield, and purity of lactosucrose reached the maximum values. However, under the temperature of 70°C, the time required for complete elution became longer, resulting in the reduction of the resolution, yield, and purity of lactosucrose. These results may be caused by the enhanced hydroxyl activity of carbohydrates after heating and the enhanced adsorption of the resin.

**Table 5:** Effect of temperature on the separation efficiency of lactosucrose

Temperature (°C)	Resolution	Yield (%)	Purity (%)
40	0.44	76.7 ± 0.3	79.1 ± 0.3
50	0.50	88.6 ± 0.2	84.9 ± 0.2
60	0.50	91.4 ± 0.2	87.4 ± 0.1
70	0.44	84.5 ± 0.2	80.2 ± 0.2

Note

Experiments were triplicated, and the results are presented as mean ± standard deviation (*SD*).

**Figure 10:**
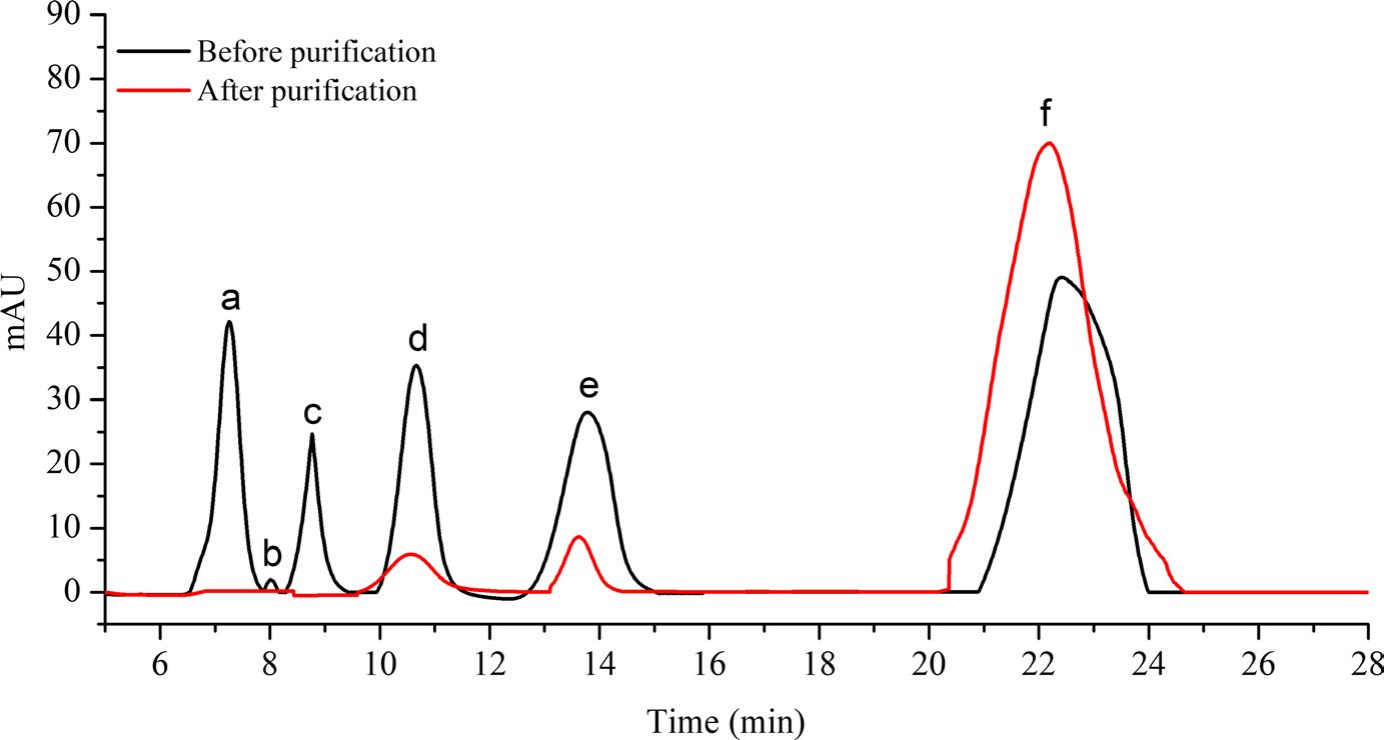
HPLC analysis of lactosucrose before and after purification. The different peaks were (a) fructose, (b) glucose, (c) gluconic acid, (d) sucrose, (e) lactose, (f) lactosucrose

According to the above results, the optimal conditions for lactosucrose separation using UBK535 calcium ester resins were at a flow rate of 0.3 ml/min and 60°C with a loading amount of 1 ml. Under this circumstance, it was found that, after purification with chromatographic separation, the resolution and yield of lactosucrose was 0.50, 91.4%, respectively, and the HPLC analysis (Figure 10) showed that the purity of lactosucrose was improved to 87.4% as compared to raw samples before separation (40.2%).

The structure of the purified lactosucrose was determined by nuclear magnetic resonance analysis. The chemical shifts of the 18 carbon atoms and protons of the compound were presented in Table 6. The ^13^C NMR spectrum showed the lower chemical shift (δ_C_) of the three terminal carbon atoms, which are 103.21, 92.11, and 104.12 ppm, respectively. In addition, the chemical shifts (δ_C_) of the four methylene carbons are relatively high, which are 61.55, 60.11, 61.54, and 62.99 ppm, respectively. The ^13^H NMR spectrum indicated that the structure of compound has three hexoses and contains two protons attached to the terminal carbon atoms with chemical shifts (δ_H_) of 5.87 and 4.76 ppm, respectively. Based on the above results, the chemical name of the compound was identified as O‐β‐d‐galactosyl‐(1,4)‐O‐α‐d‐glucosyl‐(1,2)‐β‐d‐fructose, that is, lactosucrose. The data obtained in this study are consistent with previous studies on ^13^C and ^1^H NMR analysis of lactosucrose (Han et al., 2009).

**Table 6:** Chemical shifts in ^1^H and ^13^C spectra of the lactosucrose produced from lactose and sucrose by immobilized bi‐enzymes

Group	Carbon atoms	δ_C_	δ_H_
Galactose	1	103.21	4.56
	2	71.32	3.71
	3	72.61	3.79
	4	68.77	4.01
	5	75.69	3.81
	6	61.55	3.91/3.88
Glucose	1′	92.11	5.55
	2′	72.66	3.81
	3′	71.55	4.02
	4′	78.69	3.92
	5′	71.68	4.21
	6′	60.11	4.12/4.09
Fructose	1″	61.54	4.06/4.01
	2″	104.12	–
	3″	77.12	4.58
	4″	74.41	4.43
	5″	82.32	4.14
	6″	62.99	4.07/4.01

## 4 CONCLUSION

In this study, purified β‐fructofuranosidase and glucose oxidase were co‐immobilized by the sol–gel encapsulation for the production of lactosucrose. After co‐immobilization, the enzymes retained 85.39% of their activity and had a specific activity of 96.3 U/mg. The immobilized bi‐enzymes showed good operational stability, maintaining over 78.5% of their initial activity after 15 consecutive reuses. The conditions for lactosucrose production were optimized as substrate ratio of 1:1 (w/w), substrate concentration of 20% (w/v), treatment time of 8 hr, pH 6.5, temperature at 40°C, enzyme concentration of 1.0 mg/g·sol, and β‐fructofuranosidase‐to‐glucose oxidase ratio of 3:1 (w/w). Under the optimized conditions, the yield of lactosucrose synthesized by immobilized bi‐enzymes was improved to 160.8 g/L, which was relatively higher than reported results. The prepared lactosucrose was further purified by Diaion UBK535 calcium ester resin, and the optimized conditions for purification of lactosucrose were obtained. The purity of lactosucrose was improved from 40.2% to 87.4% after purification. And the structure of the purified lactosucrose was confirmed by nuclear magnetic resonance.

Considering these results, the co‐immobilized β‐fructofuranosidase‐glucose oxidase by sol–gel encapsulation showed great industrial application potential in continuous lactosucrose production in advantages of high stability and reusability, and the as‐prepared lactosucrose with high purity can be applied to many kinds of food as functional additives.

## CONFLICTS OF INTEREST

There are no conflicts of interest to declare.

## ETHICAL STATEMENTS

This study does not involve any human or animal testing.
